# Erreichbarkeitspotenziale als Wachstumsimpuls von Mittelzentren zwischen Metropolregionskernen

**DOI:** 10.1007/s00548-022-00811-4

**Published:** 2022-10-14

**Authors:** T. Ramms, J. Wedemeier

**Affiliations:** 1 regecon Gesellschaft für regional-wirtschaftliche Forschung und Beratung mbH, Bahnhofstraße 12, 21255 Tostedt, Deutschland; 2 Forschungsbereich „Räumliche Ökonomik“, Hamburgisches WeltWirtschaftsInstitut (HWWI), Fahrenheitstr. 1, 28359 Bremen, Deutschland

**Keywords:** Erreichbarkeitspotenziale, Mittelzentren, Zentrale-Orte-Konzept, Verflechtungsraum, Raumplanung, Accessibility potential, Medium-sized centers, Central place theory, Rural suburban zone, Spatial planning

## Abstract

Die Entwicklung der Teilräume von Metropolregionen fällt heterogen aus. Während die Kernstädte, ihr engerer Verflechtungsraum mit weiteren Bevölkerungszuwächsen rechnen können, müssen sich vor allem die Zwischenlagen auf weitere Wachstumsveränderungen einstellen. Der Artikel beschreibt diese Lage von Mittelzentren zwischen Metropolregionen und deren Bedeutung als Impulsgeber für das demografische und wirtschaftliche Wachstum. Anhand von Standorten der norddeutschen Metropolregionen Hamburg, Nordwest (Bremen-Oldenburg) sowie Hannover-Braunschweig-Göttingen-Wolfsburg werden Erreichbarkeitspotenziale berechnet, um die relative Zentralität von Mittelzentren zu beschreiben. Die Mittelzentren weisen dabei teilweise eine höhere Zentralität auf als die Metropolregionen selbst und tragen zur Stabilisierung der regionalen Entwicklung in den Zwischenräumen bei. Aus dieser Beobachtung lassen sich drei Schlussfolgerungen ableiten: Erstens, dass es teilweise zu Konzentrationsprozessen innerhalb der ländlich-liegenden Gemeinden und Städte kommen kann. Zweitens, dass es eine Notwendigkeit für die Flexibilisierung des Zentrale-Orte-Konzepts gibt und drittens, dass der räumlichen Planung in Zusammenarbeit mit der Regionalpolitik eine wichtige Rolle zukommt, um diese Zentren zu stärken.

## Abstract (en)

The development of the sub-areas of metropolitan regions is heterogeneous. Although the core cities can count on further population growth, the intermediate centers in particular have to adapt to further changes in growth. The article describes the location of medium-sized centers between metropolitan regions and their importance as a driving force for demographic and economic growth. Accessibility potentials are calculated on the basis of locations in the northern German metropolitan regions of Hamburg, Nordwest (Bremen-Oldenburg), and Hanover-Braunschweig-Göttingen-Wolfsburg, in order to describe the relative centrality of medium-sized centers. Sometimes, the medium-sized centers are more central than the metropolitan regions themselves and contribute to the stabilization of regional development in the spaces. Three conclusions can be drawn from this observation: first, that concentration processes can occur within rural communities and towns; second, that there is a need for flexibility in the concept of central places; and third, that spatial planning in cooperation with regional policy plays an important role in strengthening these centers.

## Entwicklung und Funktion der Mittelzentren

Die Entwicklung der Teilräume von Metropolregionen fällt heterogen aus. Während die Kernstädte und ihr engerer Verflechtungsraum mit weiteren Bevölkerungszuwächsen rechnen können, müssen sich hingegen vor allem die Zwischenlagen auf sehr heterogene Entwicklungspfade einstellen (Körner-Blätgen und Sturm 2016; Kruse und Wedemeier 2021; Stiller et al. 2011; Teuber und Wedemeier 2019). So können ein Wachstum von zentralen Orten einerseits und eine Schrumpfung von Dörfern und Streusiedlungen andererseits sehr nahe beieinanderliegen (Kruse und Wedemeier 2021; Teuber und Wedemeier 2019). Die Wachstumsausnahmen kleiner und mittlerer Städte in Zwischenlagen können zur Stabilisierung dieser Räume beitragen, indem sie als Zentren nicht nur die Aufgabe übernehmen, die Daseinsvorsorge im ländlichen Raum im Sinne der Raumordnung zu sichern, sondern auch zu wesentlichen Treibern der regionalökonomischen Entwicklung werden (Frei et al. 2018). Mittelzentren wird in der Landes- und Raumplanung daher eine normativ „stabilisierende“ Bedeutung zugeschrieben, fungieren sie doch für ländliche Regionen als wirtschaftliche Impulsgeber (Priebs 2019, S. 185). Allerdings stellt die Förderung von neuen Entwicklungsperspektiven wie etwa Dienstleistungen, Logistik und anderen überregionalen wirtschaftlichen Schwerpunkten außerhalb von Oberzentren und speziell festgelegter Regionen kein orginäres Ziel der Landesplanung dar. Stattdessen steht sie für eine Sicherung der etablierten Struktur der Zentren (und der Bewahrung von landwirtschaftlichen Bedingungen) und schränkt somit die Aktivierung neuer wirtschaftlichen Potenziale ein (Müller und Rohr-Zänker 2003). Beispielsweise findet das europaweite „Wachstum der ländlichen Zwischenräume“ in der Raumordnungspraxis in Deutschland kaum Erwähnung (Sinz 2002). Auch zeigt ein aktuelles Beispiel, dass die Regionalplanung flexible Verfahren benötigt, um auf den Klimawandel Antworten finden zu können (BBSR 2022a; BMVI 2017). Zwar findet eine Aktualisierung bzw. die Neuaufstellung von regionalen Raumordnungsprogrammen laufend statt, allerdings kann der Prozess Jahre einnehmen, ohne Berücksichtigung der Vorlaufzeit der Neuaufstellung selbst (vgl. z. B. NMELV 2022). Die Chance, durch das flexible und zeitnahe Aufgreifen neuer Entwicklungstrends Wachstumspotenziale zu realisieren, ist grund- und mittelzentralen Orten damit vielfach durch raumordnerische Vorgaben verwehrt.

Damit droht eine Zementierung und Lock-in-Situation des regionalökonomischen Status-quo, was auch das Risiko einer Schrumpfung bestimmter Regionen impliziert (Pflüger 2019). Dies wird auch in einem Ansatz von Milbert (2017) diskutiert und herausgestellt, dass eine Notwendigkeit hinsichtlich neuer Raumtypen in der Mobilitätsforschung bestehen. Dem gegenüber ist es ein Kernanliegen des Bundes und der EU-Politik einen politisch-sozialen Zusammenhalt, Gleichheit der Lebensverhältnisse sowie für ein wirtschaftliches Zusammenwachsen der Regionen beizutragen (IWH 2019; Rauhut und Komornicki 2015). Eine Lösung kann darin bestehen, die Zentralität von Mittelzentren dynamisch zu bewerten.

In der vorliegenden Untersuchung erfolgt eine Erreichbarkeitsanalyse auf der Basis von Reisezeiten zwecks der Diskussion der Lage von Mittelzentren zwischen Metropolregionen. Das Bundesinstitut für Bau‑, Stadt- und Raumforschung (BBSR) verfolgt eine ähnliche Strategie und ermittelt im Kontext der Raumbeobachtung Verflechtungen um ein Mittelzentrum herum, welche sich in ihrer Abgrenzung an den Entfernungen, Lagebeziehungen und Verkehrsanbindungen zwischen den einzelnen Gemeinden orientieren. Die räumliche Zuordnung dieser Gemeinden zu einem Versorgungsbereich erfolgt hier ebenfalls durch die Messung der Erreichbarkeit durch die Pkw-Fahrzeit (BBSR 2022b). Ziel in diesem Aufsatz ist es nicht, eine neue Berechnungsmethode abzubilden, sondern die zentrale Mittellage in den Diskurs der Raumplanung zu stellen. Durch diese Erreichbarkeitsanalyse sollen zwei Forschungsfragen diskutiert und für die weitere wissenschaftliche Diskussion angeregt werden:

1. Forschungshypothese: Herausstellung der Zwischenräume von Oberzentren bzw. Metropolregionskernen als Entwicklungsräume der Regionalplanung;
2. Forschungshypothese: Dass es einer Flexibilisierung der Regionalplanung bedarf, um in den Regionen Antworten auf stetig dynamische Veränderung zu finden.

Für die Untersuchung werden die Bevölkerungs- und Beschäftigtenpotenziale anhand eines Beispiels der drei niedersächsischen Mittelzentren Rotenburg (Wümme), Soltau und Vechta, der Kernstädte der zugehörigen norddeutschen Metropolregionen Hamburg, Nordwest (Bremen-Oldenburg) und Hannover-Braunschweig-Göttingen-Wolfsburg betrachtet. Dabei wird deutlich, dass Mittelzentren im Zwischenraum von Metropolregionen erhebliche Erreichbarkeitspotenziale aufweisen und wesentlich zur Stabilisierung hinsichtlich demografischer und wirtschaftlicher Entwicklung beitragen können. Anhand der Ergebnisse wird die regionalökonomische Bedeutung der mittelzentralen Städte und deren raumordnerischen Festsetzungen bewertet.

Der Artikel ist wie folgt aufgebaut: Zu Beginn werden Untersuchungsraum und Methodik dargestellt, mit der die räumliche Entwicklung und Erreichbarkeitspotenziale untersucht werden. Darauf folgt die Ergebnisdarstellung. Anschließend werden die Ergebnisse diskutiert und eingeordnet. Der Artikel schließt mit einer Zusammenfassung und zeigt die Grenzen der Untersuchung auf.

## Räumliche Entwicklung und Erreichbarkeitspotenziale

### Der Untersuchungsraum für die Analyse

Als Untersuchungsraum wurden die Zwischenräume der drei Metropolregionen Norddeutschlands – Metropolregion Hamburg, Nordwest und Hannover-Braunschweig-Göttingen-Wolfsburg – als Beispiel für die Untersuchung herangezogen. Die Städte Rotenburg (Wümme), Soltau und Vechta befinden sich in einer Entfernungsdistanz von durchschnittlich 54,4 km zu den Großstadtkernen der betrachteten Metropolregionen. Die räumliche Entfernung der betrachteten Mittelzentren zu den nächsten Oberzentren sowie Kernen der Metropolregionen ist als hinreichend einzuschätzen (vgl. Zentrale Orte Monitoring des BBSR 2022b). Im Umkreis von etwa 45,6 km um Soltau befinden sich die Oberzentren Celle und Lüneburg, im Umkreis von rund 49,1 km um Vechta liegen die Oberzentren Osnabrück und Oldenburg, im Umkreis von etwa 47,4 km um Rotenburg (Wümme) befinden sich die Oberzentren Hamburg-Harburg und Bremen. Dabei wurde das Oberzentrum Osnabrück zunächst in die Überlegung der Analyse einbezogen; jedoch stellt die Stadt Osnabrück selbst keinen Kern einer Metropolregion dar und wurde daher ausgenommen.

**Abb. 1:**
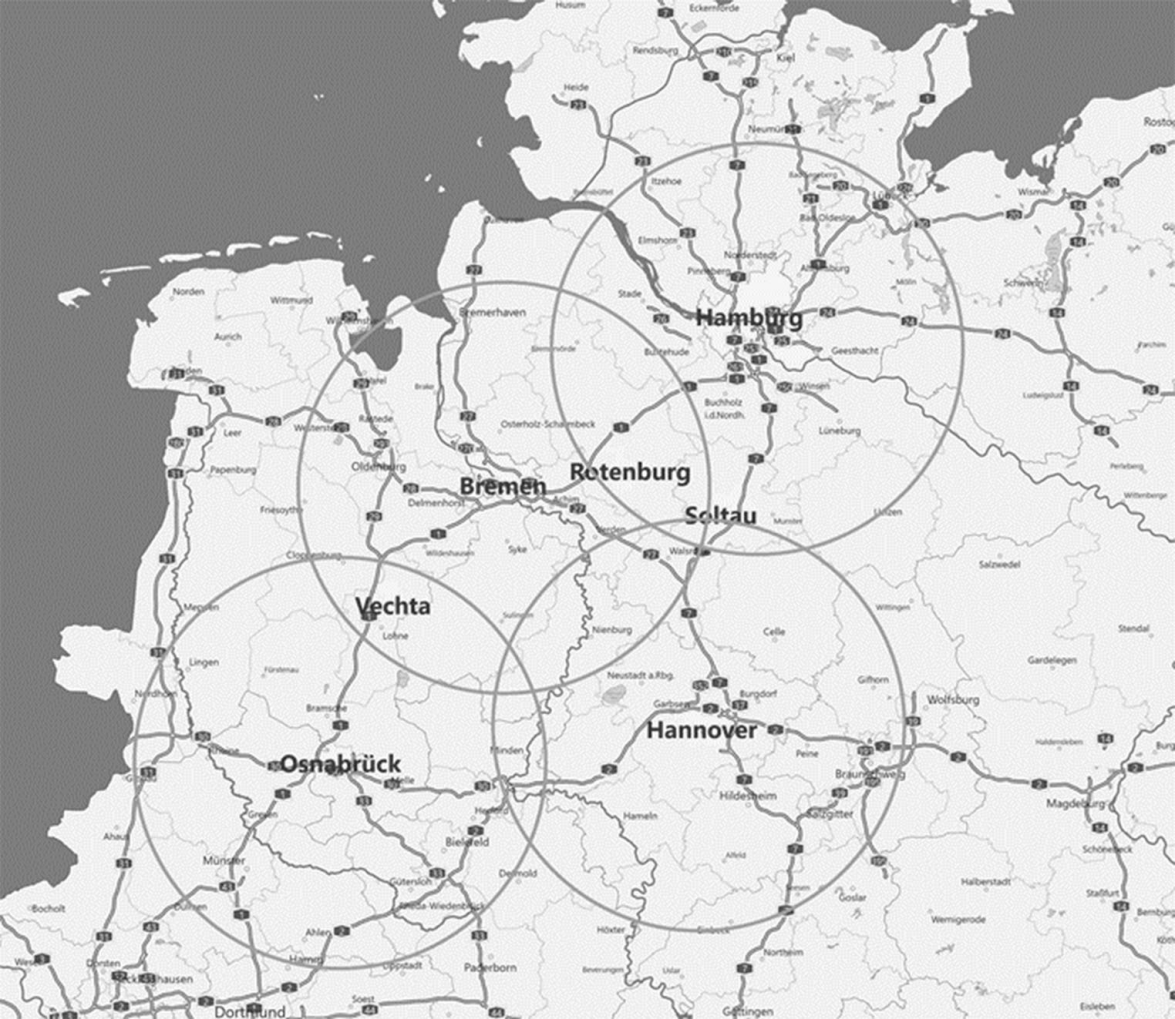
Entfernung der Mittelzentren zu den nächsten Kernen der Metropolregionen. (Quelle: Eigene Darstellung)

Die Städte Rotenburg (Wümme), Soltau und Vechta nehmen in den Grenz- bzw. Überlappungsbereich der drei Metropolregionen eine exponierte Stellung ein. Diese ist auf die verkehrsgünstige Lage zurückzuführen. Die Autobahnen ermöglichen eine schnelle Anbindung zu den Oberzentren und Kernen der Metropolregionen. Im Schienenverkehr liegt die Stadt Soltau im Schnittpunkt der Nebenstrecken Hamburg-Buchholz-Hannover und Bremen-Langwedel-Uelzen. Vechta wird in einer Nebenstrecke mit den Zentren Bremen-Osnabrück direkt verbunden. Einzig die Stadt Rotenburg ist direkt in einer Hauptstrecke mit Bremen-Hamburg und in einer Nebenstrecke mit Verden (Aller) verbunden (vgl. Abb. 1).

Die sozioökonomischen Kerndaten zeigen einen ähnlich günstigen Rahmen für die Entwicklung der Mittelzentren auf. Als Stichjahr wurde 2019 gewählt, um mögliche COVID-19-bedingte Effekte auszuschließen. Mit rund 21.000 Einwohnern sind die beiden Mittelzentren Rotenburg (Wümme) und Soltau in etwa vergleichbar. Die Städte weisen alle eine positive Bevölkerungsentwicklung auf, allerdings verlief die Entwicklung in dem betrachteten Jahren 2011–19 für die Stadt Soltau nicht so dynamisch wie für die anderen beiden Städte. Die Beschäftigungsentwicklung ist hingegen sehr heterogen (12–21 % in den Jahren 2011–2019) und nicht immer überdurchschnittlich (Rotenburg/Wümme, Vechta). Der überdurchschnittliche Beschäftigtenbesatz der Städte von rund 60 % korrespondiert mit einem hohen Einpendlerüberschuss. Der Beschäftigtenbesatz ist definiert als die Anzahl der sozialversicherungspflichtig Beschäftigten je 1000 Einwohner einer Gebietskörperschaft. Die Mittelzentren zeigen überdurchschnittlich viele Arbeitsplätze auf und sind somit Arbeitsort und wirtschaftliches Zentrum der Region (Tab. 1).

**Tab. 1:** Ökonomische Kern- und Pendlerdaten der Mittelzentren Rotenburg (Wümme), Soltau und Vechta 2019. (Quelle: Bundesagentur für Arbeit (2021); Landesamt für Statistik Niedersachsen (2021a, b), Statistische Ämter des Bundes und der Länder (2021a, b, c, d), eigene Berechnungen)

Gemeinde	Rotenburg (Wümme)	Soltau	Vechta	Niedersächsischer Durchschnitt*
Bevölkerung	21.956	21.268	32.863	19.640
Entwicklung der Bevölkerung, 2011–2019	4,0 %	0,1 %	8,4 %	2,8 %
SV-Beschäftigte am Arbeitsort	13.787	12.777	19.999	7390
SV-Beschäftigte am Wohnort	9258	8470	14.248	7726
Entwicklung der SV-Beschäftigten, 2011–2019	12,0 %	21,0 %	13,7 %	17,6 %
Beschäftigtenbesatz	62,8 %	60,1 %	60,9 %	37,6 %
Einpendler	8644	7703	12.582	–
Auspendler	4116	3402	6839	–
Einpendler‑/Beschäftigtenquote	62,7 %	60,3 %	62,9 %	–
Auspendler‑/Einwohnerquote	19 %	16 %	21 %	–
Auspendler‑/Beschäftigtenquote	44,5 %	40,2 %	48,0 %	–
Pendlersaldo	4528	4301	5743	–
Auspendler nach Hamburg	356	328	67	–
Auspendler nach Bremen (Stadt)	654	54	176	–
Auspendler nach Hannover	40	138	41	–

Hinweis: *SV* sozialversicherungspflichtig, Beschäftigtenbesatz = (SV-Beschäftigte am Arbeitsort/Bevölkerung)

### Methodische Einordnung des Konzepts Erreichbarkeit

Bei einer Bewertung der Zentren von Metropolregionen kann das Konzept der Erreichbarkeit als Anknüpfungspunkt für Wachstums- oder Standortanalysen sowie Vergleichen von Städten und Regionen dienen (Ettema und Timmermans 2007; Handy und Niemeier 1997; Hansen 1959; Weibull 1976, 1980). Trotz der weitverbreiteten Umschreibung von Erreichbarkeit als „nearness, proximity, ease of spatial interaction, potential of opportunities for interaction“ (Weibull 1980, S. 54) bleibt eine definitorische Diffusität des Konzepts bis heute bestehen (Hesse et al. 2012). Als Forschungsansatz wird Erreichbarkeit in einer Vielzahl von regional-, raum-, und verkehrswissenschaftlichen Publikationen herangezogen. Evangelinos und Ebert (2011) nutzen das Konzept der Erreichbarkeit etwa zur Messung von qualitativen Unterschieden der Verkehrsinfrastruktur. López et al. (2008) ziehen anhand von großen Infrastrukturinvestments und der Erreichbarkeitspotenziale eine Bilanz zur Kohäsionspolitik und kommen zum Fazit, dass Investitionen in die Verkehrsinfrastruktur einen wesentlichen Beitrag zur Raumentwicklung leisten. In Cheng und Bertolini (2013) wird die relative, räumliche Distanz und Funktion von Arbeitsmärkten für den Großraum Amsterdam in den Niederlanden durch ein GIS-Model aufgezeigt. Gather (2003) ermittelt mithilfe von Erreichbarkeitsanalysen Einzugsbereiche und Einwohnerpotenziale, um Empfehlungen für die Anpassungen des zentralörtlichen Systems von Mittelzentren in Thüringen zu geben. Die Messung von Erreichbarkeit in Metropolregionen wird in Levine et al. (2012) diskutiert. Ziel in diesem Aufsatz ist es nicht, eine neue Berechnungsmethode abzubilden, sondern die zentrale Mittellage in den Diskurs der Raumplanung zu stellen und somit eine Ableitung hinsichtlich der Bedeutung der Mittelzentren auch im Vergleich mit Oberzentren und Metropolregionskernen abbilden zu können. Ein neuer Ansatz zur Berechnung von Erreichbarkeitspotenzialen ist daher nicht das primäre Ziel, sondern mithilfe der Potenziale werden die Zwischenräume der Zentren, *hier* von Metropolregionen, neu bewertet.

Zur Quantifizierung der Erreichbarkeitspotenziale wurde in diesem Beitrag ermittelt, wie viele Einwohner bzw. sozialversicherungspflichtig Beschäftigte die drei untersuchten Mittelzentren Rotenburg (Wümme), Soltau und Vechta in einer bestimmten Pkw-Fahrtzeit erreichen können. Die so ermittelte relative Fahrzeit wurde herangezogen, da die Betrachtung des Raums in Zeiteinheiten (Isotimen) insofern der absoluten Entfernung vorzuziehen ist, als dass in einem Hochlohnland wie Deutschland die Transportkosten stärker von den Opportunitätskosten der Zeit, in der der Raum überwunden werden kann, als von den streckenbezogenen Fahrkosten (z. B. Kraftstoff) determiniert werden. Als Vergleichsgrößen wurden auch die Bevölkerungs- und Beschäftigtenpotenziale für die drei Großstädte Bremen, Hamburg und Hannover ermittelt. Die Berechnung der in einer bestimmten Fahrzeit erreichbaren Bevölkerungs- und Arbeitsplatzpotenziale erfolgte durch Summation der Einwohner- bzw. Beschäftigtenzahlen der Gemeinden für all jene Kommunen, die innerhalb dieser Fahrzeit (arithmetisches Mittel aus minimaler und maximaler Fahrzeit) den jeweiligen Zielort erreichen können.

In die Stichprobe miteinbezogen wurden alle Gemeinden der Bundesländer Niedersachsen, Nordrhein-Westfalen, Schleswig-Holstein und Mecklenburg-Vorpommern, aus denen man den entsprechenden Zielort in bis zu zwei Stunden Fahrtzeit erreicht. Die Ermittlung der Fahrzeiten nach Rotenburg (Wümme), Soltau, Vechta, Bremen, Hamburg und Hannover erfolgte mithilfe des Online-Kartenprogramms Google Maps (Google LLC, Mountain View, CA, USA). Als Startpunkt wurde jeweils das geografische Zentrum der untersuchten Gemeinden verwendet. Die Fahrzeit wurde bei Google Maps als Stichprobe zur Hauptverkehrszeit um 7 Uhr morgens am Montag, den 11. Februar 2019 gezogen. Dabei wurden jeweils zwei Extrema, die minimale und maximale Fahrzeit, betrachtet und anschließend der Mittelwert aus beiden ermittelten Fahrzeiten für die jeweiligen Gemeinden gebildet. Als vereinfachende Modellannahme wurde unterstellt, dass das gesamte Bevölkerungs- bzw. Beschäftigtenpotenzial einer Kommune – unabhängig von ihrer Größe innerhalb von zehn Minuten Fahrzeit erreicht werden kann.

Zur Berechnung der Erreichbarkeitspfade über die Fahrzeit zwischen verschiedenen Gemeinden, gibt es auch andere Tools, wie der von Weber et al. (2022) entwickelten Stata-Befehl *georoute* (*HERE WeGo maps*) zur Fahrzeitberechnung (StataCorp LLC, College Station, Texas, USA). Dieser Befehl ermöglicht es dem Benutzer, die Zeit abzurufen, die er benötigt, um mit dem Auto zwischen zwei Adressen oder geografischen Koordinaten unter normalen Verkehrsbedingungen zu reisen (z. B. González-Chapela und Ortega-Lapiedra 2021). Die Google-Maps-Fahrzeiten lassen hingegen tageszeitabhängige Fahrzeitenabfragen zu (in der Annahme von auftretendem Pendlerverkehr), sodass die Analyse hier mit Google durchgeführt wurde. Auch hat die wissenschaftliche Relevanz von Google Fahrzeitenberechnungen im Rahmen von Analysen der COVID-19-Pandemie zugenommen.

Auf eine Betrachtung der Fahrzeiten mit dem Schienenverkehr/ÖPNV wird hier verzichtet, da ausgehend von den geografischen Startpunkten der kleineren Gemeinden oftmals keine sinnvolle Verbindung in den Zielort besteht. Die Pkw-Fahrzeiten werden daher als Proxy der Erreichbarkeit herangezogen. Da nur eine Stichprobe für ein definiertes Datum vorliegt, können eventuelle Ausreißer (durch Baustellen, Straßensperrungen usw.) nicht ausgeschlossen werden. Zudem findet durch die Wahl der Mindestfahrzeit als Ausschlusskriterium die Untersuchung teils über dem Gebiet der Metropolregionen statt oder schließt nicht alle Kommunen der Metropolregionen mit ein. Das Zeitfenster von zwei Stunden wurde herangezogen, um eine Grenze der maximalen Pendlerdistanzen zu definieren. Die Bundesagentur für Arbeit definiert Nahpendeln aus einem Kreis zu den zehn unmittelbar nächsten Kreisen der Nachbarschaft (etwa 150 km) (Bundesagentur für Arbeit 2021).

### Untersuchungsergebnisse der Erreichbarkeitsanalyse

**Abb. 2:**
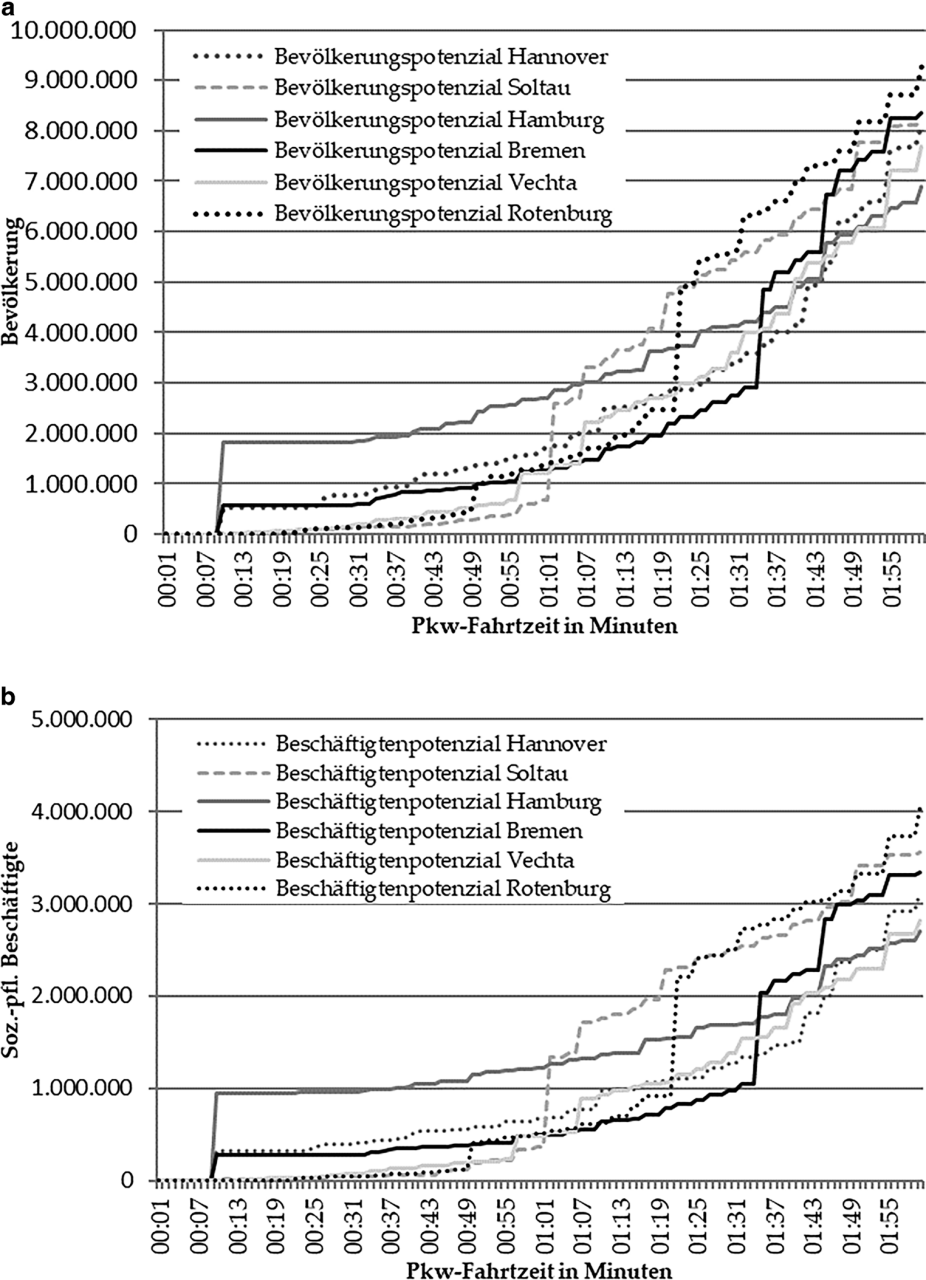
Erreichbarkeit von (**a**) Bevölkerungs- und (**b**) Beschäftigtenpotenzialen. (Quelle: Google Maps (2021), IT.NRW (2021), Landesamt für Statistik Niedersachsen (2021a, b), Statistische Ämter des Bundes und der Länder (2021b), Statistisches Bundesamt (2021), eigene Berechnungen)

Die Abb. 2 zeigt jeweils die in einer bestimmten Fahrtzeit erreichbare Bevölkerung und Beschäftigte der untersuchten Mittelzentren und Städte der Metropolregionen Hamburg, Nordwest (Bremen-Oldenburg) und Hannover-Braunschweig-Göttingen-Wolfsburg. Der Verlauf der Potenziale ist stufenförmig. An der Stufenintensität ist deutlich erkennbar, zu welchem Zeitpunkt große Ballungsräume (Großstädte) bzw. bevölkerungsreiche Gemeinden aus den drei untersuchten Mittelzentren Rotenburg (Wümme), Soltau und Vechta erreicht werden.

Erwartbar ist, dass bei geringer Fahrtzeit zunächst die Großstädte das größere Bevölkerungs- und Beschäftigtenpotenzial aufweisen. Dies wird bedingt durch ihre inhärenten Potenziale aufgrund der Bevölkerungsstärke und durch die Konzeption des Modells, bei dem keine innerstädtischen Entfernungen berücksichtig werden.

Betrachtet man die Bevölkerungspotenziale, ist erkennbar, dass die drei Metropolen bei einer Fahrtzeit von bis zu 45 min die meiste Bevölkerung erreichen, analog zu ihrer Einwohnerzahl. Bei voranschreitender Fahrtzeit schließen die Mittelzentren zu den Kernen der Metropolregionen auf und weisen ab einer Stunde Fahrtzeit im direkten Vergleich ein höheres Bevölkerungspotenzial auf; Rotenburg schließt mit Bremen auf (bei Minute 49), Vechta mit Hannover und Soltau übersteigt Hamburgs Erreichbarkeitspotenzial (beide ab 67 min Fahrtzeit).

Diese sprunghaften Anstiege der Mittelzentren geben wieder, zu welchem Zeitpunkt jeweils Metropolstädte erreicht werden können und visualisieren das Potenzial der untersuchten Mittelzentren. Bei einer Fahrtzeit zwischen 75 und 105 min zeigen Soltau und Rotenburg ein deutlich höheres Bevölkerungspotenzial als alle anderen untersuchten Städte. Eine Bevölkerung von 5 Mio. Einwohnern kann die beiden Städte mit einer Fahrt von bis zu einer Stunde und 20 min erreichen. Der sichtbare Vorsprung verdeutlicht die zentrale Lage der beiden Mittelzentren zwischen den Großstädten und damit einhergehende hohe Erreichbarkeit für Menschen der gesamten Metropolregion. Erst bei einer Fahrtzeit darüber hinaus ermöglicht die Lage Bremens der Stadt ein ähnlich hohes Bevölkerungspotenzial. Ab zwei Stunden Fahrt holen Vechta und Hannover auf, Hamburg bleibt im Gesamtpotenzial leicht zurück.

In der Analyse wird angenommen, dass eine Fahrtzeit von bis zu zwei Stunden für die Inanspruchnahme zentralörtlicher Einrichtungen in Betracht genommen wird. Vechta besitzt in dieser Zeitspanne ein geringeres Bevölkerungspotenzial als die vergleichbaren Mittelzentren Rotenburg (Wümme) und Soltau, entfaltet jedoch ein erhebliches Bevölkerungs- und Beschäftigtenpotenzial bei einer Fahrtzeit von 2 h und 15 min, wenn der Großstadtkern Hamburg erreicht werden kann.

Die Veranschaulichung der Beschäftigtenpotenziale gibt ein vergleichbares Bild ab (Abb. 2). Hamburg weist aufgrund seiner eigenen Größenstruktur zunächst das größte Erreichbarkeitspotenzial auf, bis dieses von Soltau (bei 60 min Fahrtzeit) und Rotenburg (Wümme) (80 min Fahrtzeit) durch Erreichen der Großstadt Hamburg selbst übertroffen wird. Nur Bremen weist erst ab einer Fahrtzeit von Eindreiviertel Stunden ein mit den beiden Mittelzentren vergleichbares Erreichbarkeitspotenzial von 3 Mio. Beschäftigten auf.

## Berücksichtigung von Erreichbarkeitspotenzialen in der Raumordnung: Diskussion

Die Ziele und Grundsätze zur gesamträumlichen Entwicklung Niedersachsens sowie seiner Teilräume sind – wie in den anderen Bundesländern – in regelmäßig aktualisierten Landes-Raumordnungsprogrammen geregelt (LROP-VO 2017a, b). Zur Sicherung der Daseinsvorsorge im ganzen Land werden im Landes-Raumordnungsprogramm und den darauf basierenden Regionalen Raumordnungsprogrammen Zentrale Orte festgelegt, die zumeist gleichzeitig als zentrale Siedlungsgebiete innerhalb der Kommune definiert werden. Die Oberzentren sind jene, die den spezialisierten höheren Bedarf abdecken, die Mittelzentren stellen hingegen zentralörtliche Einrichtungen und Angebote zur sogenannten Deckung des gehobenen Bedarfs zur Verfügung. Die Grundzentren decken den allgemeinen täglichen Bedarf.

Die zentralen Kriterien zur Festlegung von Ober- und Mittelzentren in der LROP-VO (2017b) sind die zwei quantifizierbaren Variablen (i) Bevölkerungszahl sowie (ii) Arbeitsmarktzentralität. Weitere Kriterien des LROP sind „weiche“ Tatbestände wie (iii) positiver Wanderungssaldo, (iv) Einwohnerdichte, (v) Einrichtungen des Gesundheitswesens, (iv) überregionale Verkehrsinfrastruktur (LROP-VO 2017b). Daneben gibt es den Richtwert des räumlichen Abstands zwischen den Mittel- und Oberzentren sowie der zentralörtlichen Funktion der benachbarten Zentren (LROP-VO 2017b).

Bei den drei betrachteten Städten Rotenburg (Wümme), Soltau und Vechta handelt es sich nach den Festsetzungen der Raumordnung (LROP-VO 2017a; LK HK RROP 2015; LK ROW RROP 2017; LK VEC RROP 2019) um Mittelzentren, die gemäß des Landes-Raumordnungsprogramms Niedersachsen 2017 zentralörtliche Einrichtungen und Angebote zur Deckung des gehobenen Bedarfs zu sichern oder zu entwickeln haben. Hierzu zählen Einrichtungen der allgemeinen und beruflichen Aus- und Weiterbildung, Einrichtungen im Sozialbereich sowie größere Anlagen im Bereich von Freizeit und Sport, aber auch Einzelhandelseinrichtungen (MKRO 2016). Wesentliche explizite Festsetzungen gibt es seitens der Landes-Raumordnung für die drei Städte nicht. Explizit erwünscht ist in Abschnitt 4.1.1 Ziffer 3 LROP-VO Niedersachsen allerdings, Logistikregionen und logistische Knoten zu entwickeln.

Die Raumordnungsplanung gewährt den drei Städten Rotenburg (Wümme), Soltau und Vechta somit die gemäß der aktuellen Kriterien der LROP höchste mögliche Zentralität (LROP-VO 2017b). Den hohen Entwicklungspotenzialen der drei Mittelzentren werden die Vorgaben der Landes-Raumordnungsprogramme jedoch nicht gerecht. Dabei machen die in dieser Kurzuntersuchung dargestellten Konzentrationstendenzen deutlich, dass (i) Mittelzentren für die sozioökonomische Entwicklung großstadtferner Regionen eine große Bedeutung einnehmen können und (ii) diese Regionen ihre Entwicklungspotenziale durch die Vorgaben der Landes-Raumordnung – zumindest im Beispiel Niedersachsens – nicht in vollem Umfang ausgeschöpft werden. Entwicklungschancen für großstadtferne Regionen – insbesondere in den peripheren Teilen von Metropolregion – werden somit vergeben.

Ein Ansatz ist, um diese Lücke im Städtesystem zu schließen, das Konzept der Regiopole. Der Begriff bezeichnet Oberzentren, die sich außerhalb der Metropolregionen befinden, die Daseinsvorsorge im ländlichen Raum gewährleisten und zusätzlich eine besondere regionale Rolle spielen. Es handelt sich dabei um kleine Großstädte, die jedoch keinen Metropolstatus aufweisen können. Das Potenzial dieser Regiopole ist umso höher, je größer ihre Masse und räumliche Entfernung zu einer Metropolregion ausfällt. Im Jahr 2009 wurde das Konzept durch den Initiativkreis der Regiopole Rostock vorgestellt und Ziele definiert, u. a. die Einführung der Regiopole als neue raumordnerische Kategorie und Berücksichtigung der Rolle der Regiopole bei der Überarbeitung der raumordnerischen Leitbilder (Aring und Reuther 2008; Regiopole 2022; Weber et al. 2020). Jedoch ist der Anwendung für das gewählte Beispiel in Niedersachsen nur bedingt übertragbar, da es sich anders als bei der Regiopole Rostock um Mittelzentren handelt, welche *per se* andere Handlungsrahmen in der Regionalplanung aufweisen als ein Oberzentrum.

Um den Aspekt der sozioökonomischen Benachteiligung ländlicher Räume Rechnung zu tragen, bedarf es der Weiterentwicklung der zentralörtlichen Abgrenzungsmöglichkeiten in der Landes-Raumordnungsplanung sowie – im Vorwege – weiterer analytischer und statistischer Betrachtungen dieses Sachverhalts (Heinze 2007; Falck et al. 2008, Mose 2018). Die Raumordnungsplanung ist somit ein nicht hinreichender, aber notwendiger Teil, der räumlichen Entwicklung: Eine Lösung kann darin bestehen die Zentralität von Mittelzentren dynamisch zu bewerten und um die Entwicklungsperspektiven des Raums zu erweitern.

Zusammenfassend, durch die Erreichbarkeitsanalyse werden die zwei Forschungsfragen, welche als Hypothese aufgestellt worden sind, wie folgt beantwortet:

1. Herausstellung der Zwischenräume von Oberzentren bzw. Metropolregionskernen als Entwicklungsräume der Regionalplanung: Grund- und mittelzentrale Orte in Zwischenräume von Oberzentren bzw. Metropolregionskernen können einen Ansatz zur Stabilisierung des Zwischenraums liefern;
2. Flexibilisierung der Regionalplanung: Diese ist erforderlich, um Antworten auf eine stetig dynamische anwachsende Veränderung in den Regionen zu finden.

Einschränkungen bietet die Analyse in diesem Papier jedoch in mehrfacher Hinsicht. Zum einen bietet das Papier nur einen Einblick in die Lage von Mittelzentren anhand dreier ausgewählter Metropolregionen Norddeutschlands. In zukünftigen Arbeiten könnte man dies mit Analysetools wie mit dem Stata-Befehl *georoute *ausweiten, wenn auch es Vorteile in der Verwendung von Google, wie hier geschildert, gibt, um weitere Hinweise hinsichtlich der Zwischenräume zu erhalten. Allerdings ist für dieses gewählte Beispiel die Relevanz für die LROP bereits ausreichend gegeben und nachgewiesen. Zweitens sind Anpassungen an die Regionalplanung herausfordernd und benötigen eine lange Vorausplanung. Dies ist zum einen das Kernargument, eine Flexibilisierung zuzulassen, zum anderen ist es die Herausforderung, überhaupt eine Flexibilisierung des LROP zu ermöglichen. Schließend sind weitere Daten des Monitorings der Zentralen Orte für die Kernbewertung notwendig. Dies wurde *hier* entsprechend skizziert, und ist ein Anknüpfungspunkt für weitere Forschung. Das Papier selbst soll keine Politik- oder Handlungsanweisungen für z. B. die verkehrlich-infrastrukturelle Entwicklung geben, sondern ausschließlich hinsichtlich der Handhabung der Regionalplanung. Insoweit ist dies eine weitere Einschränkung des Artikelbeitrags.

## Fazit

Die regionale Heterogenität nimmt in den Teilräumen der Metropolregionen Hamburg, Nordwest Bremen-Oldenburg und Hannover-Göttingen-Braunschweig-Wolfsburg bei gleichzeitiger Verflechtung und Dynamik zu. Einerseits verzeichnen die peripheren Teilräume der Metropolregion aufgrund ihrer dezentralen Lage vielerorts rückläufige Bevölkerungs- und Beschäftigtenzahlen. Andererseits gibt es innerhalb dieser Räume einzelne, aktuell vielfach als Mittelzentren festgesetzte Klein- und Mittelstädte, die aufgrund ihrer insgesamt positiven Entwicklung zur Stabilisierung der Gesamträume beitragen.

Aus diesen Tendenzen lassen sich drei Kerngedanken zur Entwicklung ableiten: Erstens, dass es teilweise zu Konzentrationsprozessen innerhalb der ländlich-liegenden Gemeinden und Städte kommt. Zweitens, dass es eine Notwendigkeit für die Flexibilisierung des Zentrale-Orte-Konzepts gibt und drittens, dass der räumlichen Planung in Zusammenarbeit mit der Regionalpolitik eine wichtige Rolle zukommt, um diese Zentren zu stärken. Es wurde skizziert, dass die hier betrachteten Städte schon heute *de-facto *oberzentrale Teilfunktionen für den Raum einnehmen. Die hohen Erreichbarkeitspotenziale bei Bevölkerung und Arbeitsplätzen gehen über die Werte der Kerne der untersuchten drei norddeutschen Metropolregionen hinaus. Sie bilden damit ökonomische Räume mit Entwicklungspotenzialen, die vielfach über die Entwicklungsmöglichkeiten hinausgehen, die die gegenwärtige Festsetzung als Mittelzentren zulässt. Durch größere Spielräume bei der ökonomischen Entwicklung könnten diese kleinen und mittleren Städte jedoch zu einer Stabilisierung der peripheren Teilräume von Metropolregionen beitragen.

Anders als in der Studie des IWH, welche eine klare Fokussierung der Investitions- und Sachmittel auf wenige Großstädte fordert, um die Produktivitätsentwicklung und deren Spillover-Effekte auf die umliegenden Gemeinden zu stärken (IWH 2019), kann hier postuliert werden, auch zwischen Oberzentren bzw. Metropolregionskernen gelegene Mittelzentren im Zuge der Regionalentwicklung und Raumordnungsplanung bei der Entfaltung ihre Potenziale verstärkt zu unterstützen. Weisen doch die betrachteten Mittelzentren in diesem Papier einen überdurchschnittlichen Beschäftigtenbesatz auf, welcher sich stabilisierend – auch im Sinne gleichwertiger Lebensverhältnisse – auf die Zwischenräume auswirkt.

Offen bleibt die derzeitige Entwicklung nach der COVID-19-Pandemie, während der die physische Mobilität zurückgegangen ist, die virtuelle Mobilität dagegen stark an Bedeutung gewonnen hat, verbunden mit einem Attraktivitätsgewinn suburbaner und peripherer Räume. Angesicht der Tatsache, dass diese Entwicklung bereits mehr als zwei Jahre anhält, stellt sich die Frage, ob die dezentralen Orte in Deutschland auch langfristig gestärkt aus der Krise herausgehen, welcher sich in einem höheren Zuzug an Bevölkerung an den Rändern der Metropolen festmachen lässt (Gascon und Haas 2020; Ehlert und Wedemeier 2022). Dies kann in der nahen Zukunft zu weiteren massiven Anpassungsbedarfen der Raumplanung führen.
